# Height-related changes in forest composition explain increasing tree mortality with height during an extreme drought

**DOI:** 10.1038/s41467-020-17213-5

**Published:** 2020-07-07

**Authors:** Nathan L. Stephenson, Adrian J. Das

**Affiliations:** U.S. Geological Survey, Western Ecological Research Center, Three Rivers, CA USA

**Keywords:** Climate-change ecology, Forest ecology, Population dynamics

**Arising from** A. Stovall et al. *Nature Communications* 10.1038/s41467-019-12380-6 (2019)

Recently, Stovall et al.^1^ showed that during an extreme drought, remotely sensed mortality of tall trees was more than double that of short trees. They interpreted this to be a consequence of inherently greater hydraulic vulnerability of tall trees, and suggested that tall-tree vulnerability should generalize more broadly. Here we reassess their conclusions using contemporaneous, ground-based data from near their study sites. We find that 90% of trees belong to taxonomic groups showing declining, not increasing, mortality with height, and that the overall increase in mortality with height is instead a consequence of height-related changes in forest composition, not intrinsically greater vulnerability of tall trees. Similar mechanisms likely explain mortality patterns at Stovall et al.’s sites, and, regardless, we show that their conclusions should not be accepted in the absence of robust tests of alternative mechanisms.

Because Stovall et al.’s remote-sensing approach did not distinguish among tree taxonomic groups, they could not test plausible alternative mechanisms. For example, consider the following two scenarios, each of a drought-stricken forest comprising two species. In the first scenario, mortality of both species declines with increasing tree height. However, at any given height, species B has substantially higher mortality than species A. In addition, the relative abundance of species B increases markedly with height. The net effect is that mortality in the forest as a whole increases with height (Supplementary Table 1). But because mortality declines with height for each species individually, we must reject explanations invoking intrinsically greater drought vulnerability of tall trees.

In the second scenario, species C’s mortality declines gradually with height, but species D’s mortality increases sharply with height. Even without height-related changes in relative species abundances, mortality in the forest as a whole can increase with height, even if species D is the minority species (Supplementary Table 2). But in this scenario, we must seek mortality mechanisms that can explain opposite height-related drought responses of co-occurring tree species.

To explore whether one or both of these scenarios could explain Stovall et al.’s results, we analyzed data from 89 randomly located forest plots distributed across a 1705-ha mixed-species, old-growth forest landscape, roughly 45–65 km southeast of Stovall et al.’s study areas in California’s Sierra Nevada^2,3^. During the last year of the drought (2016, also Stovall et al.’s last year of analysis), we recorded 5855 living and dead trees ≥5 m tall belonging to 15 species, which we assigned to three groups of species (hereafter: taxonomic groups) according to magnitude of mortality during the drought^2,4–6^ (Supplementary Table 3). Height classes of individual trees (5–15 m, 15–30 m, and >30 m, following Stovall et al.) were estimated from trunk diameter using species-specific allometric equations (Supplementary Table 3). Numbers of trees alive in 2013, and 2014–2016 mortality, were calculated as described in ref. ^2^ and as summarized in “Methods” section.

When all trees were considered together, our results were similar to Stovall et al.’s: mortality of the tallest trees was ~2-fold greater than that of the shortest trees (Fig. 1a). But this simple analysis masked profound—and consequential—differences among taxonomic groups in both the magnitude of mortality and its relationship to tree height. For example, across the three height classes, mortality was low (<0.09) in angiosperms, intermediate (0.17–0.26) in non-*Pinus* conifers, and high (0.17–0.56) in *Pinus* (Fig. 1b). Within each taxonomic group, individual species had magnitudes and patterns of mortality that were largely similar to one another (Supplementary Fig. 1).

**Fig. 1:**
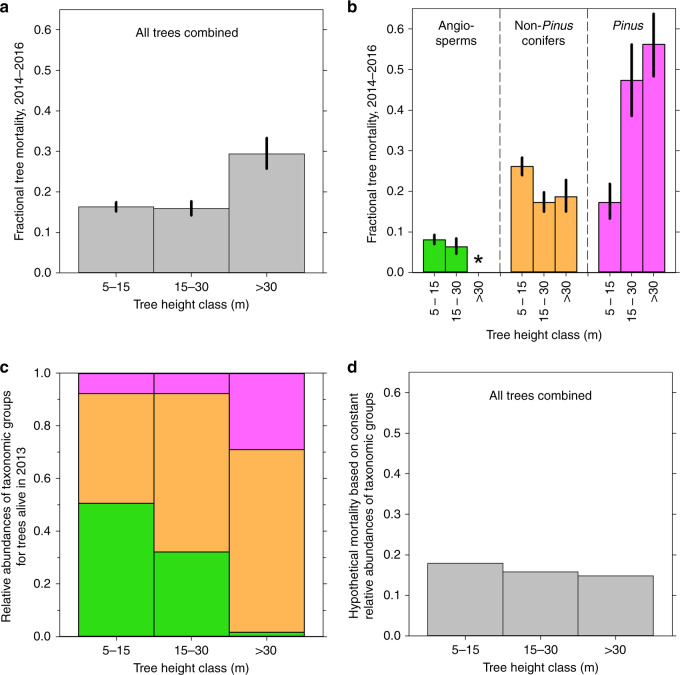
Tree mortality and relative abundances of taxonomic groups by height class during the extreme drought. **a** 2014–2016 mortality by height class, all trees combined. Bar heights show mean mortality (with 95% credible intervals) derived from the posterior distributions of parameters estimated from 45,000 Markov Chain Monte Carlo iterations (three 15,000-iteration chains), as described in Methods. **b** 2014–2016 mortality by height class for each taxonomic group (bar heights and credible intervals are as in **a**). None of the 9 angiosperms >30 m tall died, yielding 0 mortality; for reference, the asterisk indicates mortality for a larger sample (the 71 angiosperms >25 m tall). Numbers of sampled trees contributing to the other combinations of taxonomic group and height class are given in Supplementary Table 6 and Supplementary Fig. 3. **c** Relative abundances of the taxonomic groups within each height class, for trees alive in 2013 (green = angiosperms; orange = non-*Pinus* conifers; magenta = *Pinus*). **d** Hypothetical 2014–2016 mortality for all trees combined, calculated using actual height- and taxa-specific mortality values but assuming constant relative abundances of taxonomic groups across height classes (Supplementary Table 4). Because these results are hypothetical, no credible intervals are shown.

Notably, variation in mortality was greater within height classes (among taxonomic groups) than among height classes (within taxonomic groups). In addition, only 10% of trees belonged to a taxonomic group (*Pinus*) in which mortality increased with tree height. The remaining 90% belonged to groups (angiosperms and non-*Pinus* conifers) in which mortality declined slightly with height.

With increasing height, angiosperms, with their low mortality in all height classes, declined in relative abundance, whereas the intermediate-mortality non-*Pinus* conifers and high-mortality *Pinus* increased (Fig. 1c). To explore the effects of these changing relative abundances, we calculated hypothetical mortality for all trees combined, using actual height- and taxa-specific mortality values (Fig. 1b), but assuming constant relative abundances of taxonomic groups (those of the population as a whole) across the three height classes (Supplementary Table 4). Without the height-related changes in relative abundances, mortality for all trees combined would have declined slightly with tree height (Fig. 1d).

Thus, rather than being driven by increasing drought vulnerability with tree height, the observed increase in overall mortality with height (Fig. 1a) was primarily a consequence of changing taxonomic composition, similar to the first scenario. The weaker contribution of the second scenario (increasing *Pinus* mortality with height) is assessed in Supplementary Note 1, Supplementary Table 5, and Supplementary Fig. 2.

Mechanistically, the diverse height-mortality relationships of the different tree taxa—ranging from strongly declining (*Calocedrus decurrens*) to strongly increasing (*Pinus*) (Supplementary Fig. 1)—were largely a consequence of idiosyncratic host-tree selection by the different bark beetle taxa responsible for most tree mortality during the drought^2^. Furthermore, the uniquely high mortality of tall *Pinus* was unlikely to be a consequence of increased hydraulic vulnerability. Instead, the outbreaking *Dendroctonus* bark beetles responsible for killing tall *Pinus* are known to preferentially mass-attack large trees, independent of those trees’ stress^2^. Finally, besides being a consequence of the shorter stature of mature angiosperms relative to conifers^7^, the observed height-related changes in forest composition—and thus the increase in overall mortality with height—were shaped by historical contingencies (Supplementary Note 2).

Other studies conducted during the drought in forests below 2400 m (the dominant elevations at Stovall et al.’s sites) suggest that our finding of little or no role for height-related changes in tree vulnerability likely generalize to Stovall et al.’s sites, and beyond (Supplementary Note 3). First, the sharp taxonomic hierarchy in magnitude of mortality—low, intermediate, and high mortality of angiosperms, non-*Pinus* conifers, and *Pinus*, respectively—occurred broadly^2,4–6^. Second, the size-related changes in forest composition—with angiosperms declining and *Pinus* increasing in dominance with increasing tree size—also occurred broadly^8^. Finally, mortality of *Pinus* typically increased with tree size, whereas mortality of angiosperms and non-*Pinus* conifers usually showed no consistent trend or declined with size (Supplementary Note 3). Thus, rather than being unique to our study landscape, the key elements driving our conclusions appear to have occurred generally in forests below 2400 m. At higher elevations (Stovall et al.’s sites reached 3078 m), large areas are dominated by near-monocultures of *Pinus* (Supplementary Note 3). Even in high-elevation *Pinus* monocultures, we would expect mortality to increase with height, but not because tree hydraulic vulnerability increases with height. Rather, as with the low-elevation *Pinus* species, the outbreaking *Dendroctonus* bark beetles that kill high-elevation *Pinus* species preferentially mass-attack large trees, regardless of those trees’ stress (reviewed in ref. ^2^).

Our findings, and probable bias in Stovall et al.’s data (Supplementary Note 4), show we should reject Stovall et al.’s conclusion that the overall increase in mortality with height was a consequence of greater hydraulic vulnerability of tall trees. More broadly, our finding of no consistent size–mortality relationship among tree taxa during drought aligns with results of a recent multi-continent assessment^2^. Although large trees are vulnerable to many ongoing environmental changes^9^, they are not consistently the most vulnerable to drought^2^.

## Methods

### Calculations for hypothetical scenarios

In a forest comprising three taxonomic groups, the overall fractional mortality, *M*, of all trees in height class *i* is given by

1 $$M_i = (d_{i,1} + d_{i,2} + d_{i,3}){\mathrm{/}}(n_{i,1} + n_{i,2} + n_{i,3}),$$

where *n*_*i*_,_1_, *n*_*i*_,_2_, and *n*_*i*_,_3_ are the numbers of living trees in height class *i* and taxonomic groups 1, 2, and 3 at the start of the measurement period, and *d*_*i*_,_1_, *d*_*i*_,_2_, and *d*_*i*_,_3_ are the numbers of those trees that died by the end of the measurement period. Thus,

2 $$M_i = d_{i,1}{\mathrm{/}}(n_{i,1} + n_{i,2} + n_{i,3}) + d_{i,2}{\mathrm{/}}(n_{i,1} + n_{i,2} + n_{i,3}) + d_{i,3}{\mathrm{/}}(n_{i,1} + n_{i,2} + n_{i,3}),$$

3 $$\,\,\,\,\,	= (d_{i,1}{\mathrm{/}}n_{i,1})\left( {n_{i,1}{\mathrm{/}}(n_{i,1} + n_{i,2} + n_{i,3})} \right) + ( {d_{i,2}{\mathrm{/}}n_{i,2}} ) \left(n_{i,2}{\mathrm{/}}(n_{i,1} + n_{i,2} + n_{i,3})\right)\\ 	 \,\,\, + (d_{i,3}{\mathrm{/}}n_{i,3})\left( {n_{i,3}{\mathrm{/}}(n_{i,1} + n_{i,2} + n_{i,3})} \right),$$

4 $$ = m_{i,1}p_{i,1} + m_{i,2}p_{i,2} + m_{i,3}p_{i,3},$$

where *m*_*i*_,_1_, *m*_*i*_,_2_, and *m*_*i*_,_3_ are the fractional mortalities of trees in height class *i* and taxonomic groups 1, 2, and 3 over the observation period, and *p*_*i*_,_1_, *p*_*i*_,_2_, and *p*_*i*_,_3_ are the proportions of living trees in those taxonomic groups relative to all trees in height class *i* at the start of the observation period. That is, *p*_*i*_,_1_ + *p*_*i*_,_2_ + *p*_*i*_,_3_ = 1. As described in “Analysis” section (below), Eq. (4) was used to calculate hypothetical mortality of all trees within height classes for Fig. 1d, Supplementary Fig. 2, and Supplementary Tables 4 and 5.

Reflecting our paper’s main analyses, Eq. (4) assumes three taxonomic groups, but generalizes to

5 $$M_i = m_{i,1}p_{i,1} + \ldots + m_{i,x}p_{i,x},$$

where *x* represents any number of taxonomic groups ≥2. Equation (5), with *x* = 2, was used to calculate mortality of all trees within height classes for our hypothetical examples illustrating the two introductory scenarios (Supplementary Tables 1 and 2).

### Data

Details on data collection can be found in ref. ^2^; here we provide a brief summary. Within a 1705-ha forested landscape (1524–1829 m elevation), locations of our 89 0.1-ha plots were selected a priori using Generalized Random Tessellation Sampling (GRTS), which provides a spatially balanced sample that has a true probability design^10^. Within each plot, we recorded the species, trunk diameter at breast height (DBH; breast height = 1.37 m) by 5-cm classes, and condition (living or dead) of all standing conifers >0 cm DBH and all standing angiosperms ≥5 cm DBH. Each dead tree was further classified according to its foliage and fine twig retention to allow estimation of timing of death (see “Analysis” section, below). Data were collected in Microsoft Access (version 2016) using Microsoft Access forms.

Height classes of individual trees (5–15 m, 15–30 m, and >30 m, following Stovall et al.^1^) were estimated from DBH using published species-specific allometric equations (Supplementary Table 3). Sources of equations were selected for geographic proximity to our study site and for large numbers of calibration trees spanning a broad range of heights. Because DBH of our trees was recorded in 5 cm classes, DBH thresholds between height classes were assigned to the nearest 5 cm DBH (Supplementary Table 3). Although different sets of allometric equations undoubtedly would yield somewhat different species-specific thresholds among height classes, our broad conclusions would almost certainly remain unchanged; for example, the same size-related patterns of tree mortality that we found relative to tree height (Fig. 1b; Supplementary Fig. 1) are also evident relative to trunk diameter^2^.

### Analysis

Mortality was calculated as in ref. ^2^, with the exception that because our primary goal was to explore the consequences of taxonomic groups on the overall mortality of a sample, we simplified the model by excluding plot as a random effect. We first calculated the probability that a given tree died prior to 2014. The data allowing us to do this came from other plots where, extending back decades, we knew the exact years of death for 2297 standing dead trees. In the summer of 2016, we classified each of these 2297 dead trees according to the same foliage and fine twig retention classes we used in the 89 randomly located plots, allowing us to fit probability distributions (gamma distributions) for year of tree death as functions of species and foliage or twig retention class. When we lacked adequate samples for some less-common tree species, we used the probability distributions of similar taxa, as follows: *Abies magnifica* and *Torreya californica* used *A. concolor* calibration; *Pinus jeffreyi* used the combined *P. ponderosa* + *P. lambertiana* calibration; and all angiosperms used the *Quercus kelloggii* calibration. The probability distributions were then used to calculate the probability that each tree in our 89 randomly located plots died prior to the drought. Given that probability, which allows us to incorporate year-of-death uncertainty into our credible intervals, we then fit a logistic model to estimate the probability that a tree of a given taxonomic group and height class died in 2014–2016. All parameters were fit as part of Bayesian models using a Markov Chain Monte Carlo approach, with parameters having either diffuse normal or diffuse uniform (uninformative) priors, as detailed in ref. ^2^. We used three chains (unthinned) and for each chain used 20,000 iterations, with 5000 of those iterations used as burn-in. For all Bayesian analyses (including the determination of gamma distributions above), we examined traceplots of the chains to detect obvious lack of convergence, excessive autocorrelation, poor mixing, or inadequate burn-in. Convergence was also evaluated using the Gelman–Rubin diagnostic; in all cases, the Gelman-Rubin diagnostic was very near 1, indicating good convergence. Analyses were performed using R 3.6.2 with the coda 0.19.3, rjags 4.10, and R2jags 0.5.7 packages in combination with JAGS 4.3.0 software (Supplementary Code).

Within each height class *i* and taxonomic group *t*, we estimated numbers of trees that were alive in 2013 as

6 $$n_{2013,i,t} = n_{2016,i,t}{\mathrm{/}}(1 - m_{i,t}),$$

where *n*_2016,*i*,*t*_ is the actual number of living trees we recorded in height class *i* and taxonomic group *t* during our 2016 field surveys, and *m*_*i*,*t*_ is the corresponding 2014–2016 fractional mortality, estimated as described in the preceding paragraph. Estimated numbers of trees that died 2014–2016 were then calculated as

7 $$d_{i,t} = n_{2013,i,t} - n_{2016,i,t}.$$

Numbers derived from Eqs. (6) and (7) are presented in Supplementary Table 6 and Supplementary Fig. 3, and were used to calculate the various proportions, *p*_*i,t*_, shown in Supplementary Tables 4 and 5, and in associated figures.

To derive Fig. 1d, we used Eq. (4) to calculate hypothetical mortality for all trees considered together within each height class, using actual height- and taxa-specific mortality values (Fig. 1b) but assuming constant abundances of taxonomic groups (those of the population as a whole) across the three height classes for trees alive in 2013 (Supplementary Table 4). (Because they represent numbers of trees alive in 2013, these proportions differ slightly from what would be calculated from the numbers in Supplementary Table 3, which represent all living and dead trees in our sample regardless of year of death.) Similarly, we wished to explore the second introductory scenario: even though *Pinus* comprised only ~10% of trees, its increasing mortality with height must also have contributed to the overall increase in tree mortality with height shown in Fig. 1a. We thus used Eq. (4) to calculate hypothetical mortality for all trees considered together within each height class, but this time using actual relative abundances of taxonomic groups within height classes, and actual height- and taxa-specific mortality values for angiosperms and non-*Pinus* conifers, but assuming constant *Pinus* mortality (that of the *Pinus* population as a whole) across the three *Pinus* height classes (Supplementary Table 5).

### Reporting summary

Further information on research design is available in the Nature Research Reporting Summary linked to this article.

## Supplementary information

Supplementary Information

Supplementary Software

Reporting Summary

## Data Availability

The data analyzed here, originally published in support of ref. ^2^, are available in the ScienceBase repository, 10.5066/P99RNGXH.

## Code availability

The scripts used in our analyses are available under Supplementary Code.
