# Supplementary material for: Height-related changes in forest composition explain increasing tree mortality with height during an extreme drought
Source: Nat Commun. 2020 Jul 7;11:3402. doi: 10.1038/s41467-020-17213-5 (PMC7341764; doi:10.1038/s41467-020-17213-5)
Supplement: Supplementary file 1 — Supplementary Information [file 41467_2020_17213_MOESM1_ESM.pdf]

Supplementary Information

Height-related changes in forest composition explain increasing tree mortality with height during an extreme drought

Nathan L. Stephenson* & Adrian J. Das

U.S. Geological Survey, Western Ecological Research Center, Three Rivers, CA, USA

*email: nstephenson@usgs.gov

Supplementary Note 1:

When *Pinus* mortality was held constant across height classes, hypothetical mortality was reduced for trees >30 m tall (Supplementary Fig. 2, Supplementary Table 5), indicating that *Pinus*'s increasing mortality with height (Fig. 1b) indeed contributed to the high mortality of all trees >30 m seen in Fig. 1a, as in the second scenario. However, hypothetical all-tree mortality assuming constant *Pinus* mortality remained greatest, and declined less, in trees >30 m than it did when relative abundances were held constant (Fig. 1d, Supplementary Table 4). Thus, height-related changes in the relative abundances of the taxonomic groups were a stronger driver of the observed overall increase in tree mortality with height (Fig. 1a).

Supplementary Note 2:

More than a century of fire exclusion has reduced recruitment of shade-intolerant species (e.g., *Pinus ponderosa*, *Quercus kelloggii*), while allowing abundant recruitment of more shade-tolerant species (e.g., *Abies concolor, C. decurrens*)[1]. Additionally, with fire exclusion some large, old angiosperms (particularly *Q. kelloggii*) have been overtopped by conifers and killed by shading[2]. Finally, an introduced pathogen (*Cronartium ribicola*) has reduced the relative abundance of small *P. lambertiana*[3,4]. Although we cannot estimate the combined effects of these and other historical contingencies, by shaping height-related changes in forest composition they undoubtedly helped shape the observed height-related changes in overall mortality.

Supplementary Note 3:

The following supplementary information, relevant to assessing the generality of our findings, refers to three forested elevational bands: ~1500-1800 m (low-elevation forests: mostly mixed-species forests, and the forest zone that contained our study landscape), ~1800-2400 m (mid-elevation forests: mostly mixed-species forests), and ~2400-3400 m (high-elevation forests: forests often strongly dominated by single species). Details on the structure, composition, and distribution of these forests can be found elsewhere[5,6].

Importantly, even though the different forest types within the two lower elevational bands are often named for the species that typically dominate them (e.g., black oak woodland, white fir forest, ponderosa pine forest), members of all three taxonomic groups we analyzed – angiosperms, non-*Pinus* conifers, and *Pinus* – typically co-occur in most of these forest types (although angiosperm abundance also usually declines with elevation)[5,6]. The mix of taxonomic groups is relevant because the two scenarios of the main text occur in mixed forests in which different tree taxonomic groups have different height-specific mortality rates. In low- and mid-elevation forests during the drought, the sharp taxonomic hierarchy in magnitude of mortality that we found – low, intermediate, and high mortality of angiosperms, non-*Pinus* conifers, and *Pinus*, respectively – occurred broadly[7-10]. Additionally, size-related changes in forest composition – with angiosperms declining and *Pinus* increasing in dominance with size – also occurred broadly[11]. Thus, two key conditions relevant to our scenarios (the last being necessary only to the first scenario) were met broadly.

A final key condition of our two scenarios is that, even if mortality of all trees considered together increases with height, within some or all individual taxonomic groups mortality remains constant or declines with height. This was clearly the case at our low-elevation study site, where mortality increased with height for *Pinus* but declined slightly with height for angiosperms and non-*Pinus* conifers (Fig. 1b). Although sometimes hampered by small samples, other studies in low- and mid-elevation forests during the drought similarly found that *Pinus* mortality typically increased with tree size, whereas mortality of angiosperms and non-*Pinus* conifers usually showed no consistent trend or declined with size[7-10,12]. Additionally, elsewhere we analyzed thousands of trees from 12 large plots mostly in mid-elevation forests above our 1524-1829 m study landscape[7]. Although we did not include these plots in our current analyses (because we wished only to include randomly located plots), mortality of angiosperms and non-*Pinus* conifers changed little or declined with increasing tree size, as in our low-elevation plots analysed here[7].

Thus, available evidence suggests that the key elements driving our results and conclusions likely occurred broadly in low- and mid-elevation forests of the Sierra Nevada.

During the drought, tree mortality in high-elevation forests (ranging from ~2400 m to upper treeline at ~3400 m) was generally much less than in low- and mid-elevation forests, and this low mortality resulted in researchers collecting or analysing fewer ground-based data sets. Regardless, the composition of high-elevation forests allows us to frame *a priori* expectations for height-related mortality patterns there. Tree diversity declines at high elevations, often resulting in near monocultures of *Abies magnifica* or various *Pinus* species (*P. albicaulis*, *P. balfouriana*, *P. contorta*, or *P. monticola*)[5,6]. As noted in the main text, even in high-elevation *Pinus* monocultures we would expect mortality to increase with tree height during drought, but not because tree hydraulic vulnerability increases with height. Rather, the outbreaking *Dendroctonus* bark beetle species that kill *Pinus* in the Sierra Nevada preferentially mass-attack large trees, regardless of those trees' stress (reviewed in ref. 7).

In contrast, mortality of *A. magnifica* may have increased, decreased, or changed little with height, depending on the relative abundances of the three species of *Scolytus* bark beetles that typically attack *A. magnifica*: *S. ventralis*, which preferentially attacks large trees, and *S. praeceps* and *S. subscaber,* which preferentially attack small trees[7]. If relative abundances of the three *Scolytus* species were similar to those that attacked *A. concolor* during the drought[7], the near monocultures of *A. magnifica* could represent one of the few Sierra Nevada forest types for which overall mortality during the drought did not increase with tree height. However, a systematic remote-sensing bias means that Stovall et al.[13] may have spuriously detected apparent increases in mortality with height even for forest types that had gradual declines in mortality with height (Supplementary Note 4).

Supplementary Note 4:

To define individual tree crowns, Stovall et al. applied standard algorithms to their LiDAR data[13]. For open-grown trees, this approach usually successfully identifies individual crowns of trees of all heights. In contrast, when trees are crowded, in our experience the crowns

of shorter trees are often algorithmically merged with those of taller trees, or are missed entirely when the shorter trees' crowns are obscured by those of taller trees.

Indeed, Stovall et al.'s data suggest they substantially undersampled short trees. Numerically, Sierra Nevada forests are heavily dominated by small trees[11]; for example, in our plots trees 5-15 m tall were the most abundant (Supplementary Fig. 3), comprising 62% of all trees alive in 2013. In contrast, in Stovall et al.'s data set trees 5-15 m tall were the least abundant of the three height classes, comprising only 17% of trees they identified[13].

Thus, Stovall et al.'s sample of short trees could be systematically biased toward the particular subset of short trees – those growing in the less-crowded conditions that allowed successful identification by LiDAR – that had the lowest mortality. Specifically, Sierra Nevada trees growing in crowded conditions often suffer elevated mortality[14,15], both from the direct and indirect effects of competition, and from greater tree-to-tree transmission of pathogens and bark beetles (the last of which were responsible for most tree mortality during the drought[7,8]). Thus, systematic undersampling of short trees growing in crowded conditions (i.e., undersampling the high-mortality subpopulation of short trees) could introduce a bias toward finding increasing mortality with tree height, even in forest types in which mortality may have declined with height.

Supplementary Table 1. A hypothetical example of the main text's first scenario.

Height class i	Species A (taxonomic group 1)		Species B (taxonomic group 2)		Mortality, all trees in height class i
	$m_{i,1}$	$p_{i,1}$	$m_{i,2}$	$p_{i,2}$	M_i
Short	0.05	0.8	0.5	0.2	0.140
Intermediate	0.04	0.6	0.4	0.4	0.184
Tall	0.03	0.2	0.3	0.8	0.246

Notes: Symbols are defined as in Equations (4) and (5). Values of M_i were calculated using Equation (5), for $x = 2$ taxonomic groups.

Supplementary Table 2. A hypothetical example of the main text's second scenario.

Height class i	Species C (taxonomic group 1)		Species D (taxonomic group 2)		Mortality, all trees in height class i
	$m_{i,1}$	$p_{i,1}$	$m_{i,2}$	$p_{i,2}$	M_i
Short	0.05	0.7	0.05	0.3	0.050
Intermediate	0.04	0.7	0.20	0.3	0.088
Tall	0.03	0.7	0.50	0.3	0.171

Notes: Symbols are defined as in Equations (4) and (5). Values of M_i were calculated using Equation (5), for $x = 2$ taxonomic groups.

Supplementary Table 3. Characteristics of trees ≥5 m tall recorded in the 89 randomly located 0.1-ha plots.

Species	Taxonomic group	Number live+dead in 2016	Sources of height allometry‡	DBH (cm) for 5-15 m tall	DBH (cm) for 15-30 m tall	DBH (cm) for >30 m tall
Quercus kelloggii †	Angiosperm	1121	16	5-25	25-75	75-135
Umbellularia californica	Angiosperm	401	18	5-25	25-30	
Cornus nuttallii †	Angiosperm	400	20	5-35		
Quercus chrysolepis	Angiosperm	289	18	10-45	45-60	
Alnus rhombifolia †	Angiosperm	53	19*	5-15	15-55	
Acer macrophyllum †	Angiosperm	19	18	5-15	15-40	
Cercocarpus betuloides	Angiosperm	6	18**	10-25		
Aesculus californica †	Angiosperm	1	20	10-20		
Abies concolor	non-*Pinus* conifer	1672	16	10-20	20-50	50-150
Calocedrus decurrens	non-*Pinus* conifer	1231	16	10-30	30-65	65-145
Torreya californica	non-*Pinus* conifer	59	20	10-50		
Abies magnifica	non-*Pinus* conifer	2	17	5-25		
Pinus ponderosa	*Pinus*	377	16	5-20	20-40	40-150
Pinus lambertiana	*Pinus*	223	16	10-25	25-55	55-175
Pinus jeffreyi	*Pinus*	1	18	10-35	35-45	

Notes: The last three columns show the range of trunk diameters at breast height (DBH) that, based on the cited allometric equations, correspond to the three different height classes. The greatest DBH shown for each species is that of the largest tree of that species that we encountered.

† Deciduous species.

* No species-specific allometric equation found; equation for *Alnus rubra* was used.

** No species-specific allometric equation found; equation for *Quercus chrysolepis* was used.

‡ Numbers refer to publications listed in the Supplementary References.

Supplementary Table 4. Calculation of hypothetical height-specific mortality rates of all taxonomic groups combined, assuming constant abundances of taxonomic groups across the three height classes (Fig. 1d).

Height class i	Angiosperms		Non-*Pinus* conifers		*Pinus*		Mortality, all trees in height class i
	$m_{i,1}$	$p_{i,1}$	$m_{i,2}$	$p_{i,2}$	$m_{i,3}$	$p_{i,3}$	M_i
5-15 m	0.08073	**0.4071**	0.2609	**0.4943**	0.1724	**0.09863**	**0.1788**
15-30 m	0.06295	**0.4071**	0.1726	**0.4943**	0.4735	**0.09863**	**0.1576**
>30 m	0 (0.03349)	**0.4071**	0.1867	**0.4943**	0.5618	**0.09863**	**0.1477 (0.1613)**

Notes: Hypothetical values are in bold. None of the 9 angiosperms >30 m tall died, yielding 0 mortality. Thus, for reference, for the tallest angiosperms the additional value of M_i in parentheses was calculated using the associated value of $m_{i,1}$ in parentheses, which reflects a larger sample of 71 angiosperms >25 m tall. Symbols are defined as in Equations (4) and (5). Values of M_i were calculated using Equation (4).

Supplementary Table 5. Calculation of hypothetical height-specific mortality rates of all taxonomic groups combined, assuming constant mortality of *Pinus* across the three height classes (Supplementary Fig. 2).

Height class i	Angiosperms		Non-*Pinus* conifers		*Pinus*		Mortality, all trees in height class i
	$m_{i,1}$	$p_{i,1}$	$m_{i,2}$	$p_{i,2}$	$m_{i,3}$	$p_{i,3}$	M_i
5-15 m	0.08073	0.5064	0.2609	0.4154	**0.3501**	0.07813	**0.1766**
15-30 m	0.06295	0.3214	0.1726	0.6008	**0.3501**	0.07772	**0.1511**
>30 m	0 (0.03349)	0.01731	0.1867	0.6930	**0.3501**	0.2897	**0.2308 (0.2314)**

Notes: Hypothetical values are in bold. Symbols, and values in parentheses, are as in Supplementary Table 4. Values of M_i were calculated using Equation (4).

Supplementary Table 6. Numbers of trees in our 1705-ha study landscape, by taxonomic group, height class, and drought mortality status. The numbers are presented graphically in Supplementary Fig. 3.

Taxonomic group	Height class (m)	Number alive in 2016	Estimated number that died 2014-2016	Total (i.e., estimated number alive in 2013)
Angiosperms	5-15	1548	135.95	1683.95
	15-30	449	30.16	479.16
	>30	9	0.00	9.00
non-*Pinus* conifers	5-15	1021	360.42	1381.42
	15-30	741	154.61	895.61
	>30	293	67.24	360.24
Pinus	5-15	215	44.79	259.79
	15-30	61	54.85	115.85
	>30	66	84.63	150.63
Total		4403	932.65	5335.65

Notes: Numbers differ from those in Supplementary Table 3, which represent all living and dead trees in our sample regardless of year of death. Numbers alive in 2016 represent the actual numbers of living trees we encountered in our 89 randomly located plots in 2016. Estimated numbers of trees alive in 2013, and estimated numbers that died in 2014-2016, were calculated using Equations 6 and 7.

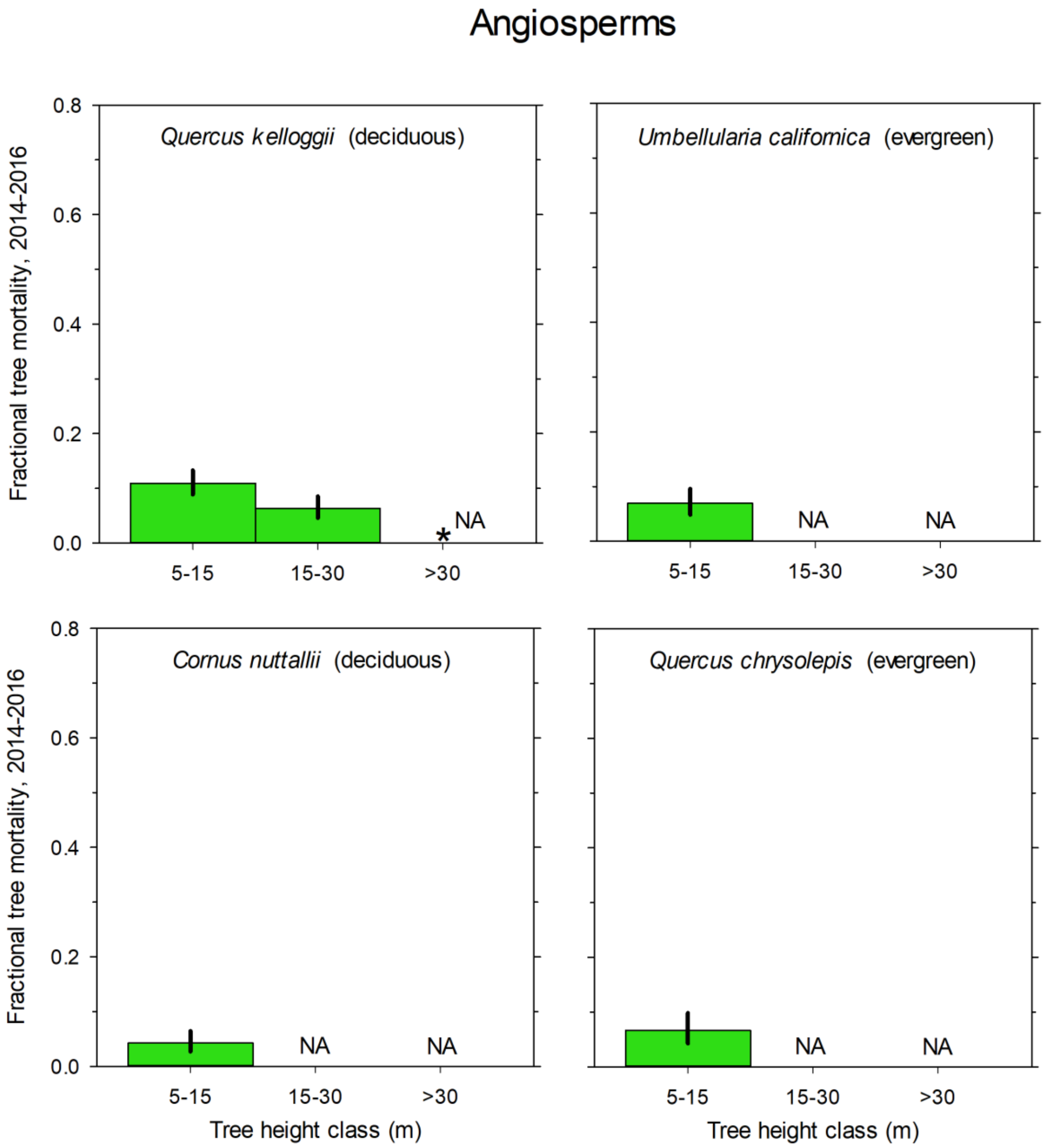

Supplementary Fig. 1 (continued on the next page): 2014-2016 tree mortality by height class for each of the eight species with >100 living and dead trees in our sample (collectively representing 97.6% of recorded trees). Bar heights show mean mortality (with 95% credible intervals) derived from the posterior distributions of parameters estimated from 45,000 Markov Chain Monte Carlo iterations (three 15,000-iteration chains), as described in Methods. Evergreen or deciduous is indicated for the four angiosperm species; all four conifer species are evergreen. Results are shown for each combination of species and height class with >50 living and dead trees in 2016; all combinations of species and height classes marked with NA had <10 trees. For reference, the asterisk at >30 m for *Quercus kelloggii* represents a 2014-2016 mortality of 0.0156 calculated for 64 *Q. kelloggii* ≥50 cm DBH (corresponding to *Q. kelloggii* ≥25 m tall), from (ref. 7).

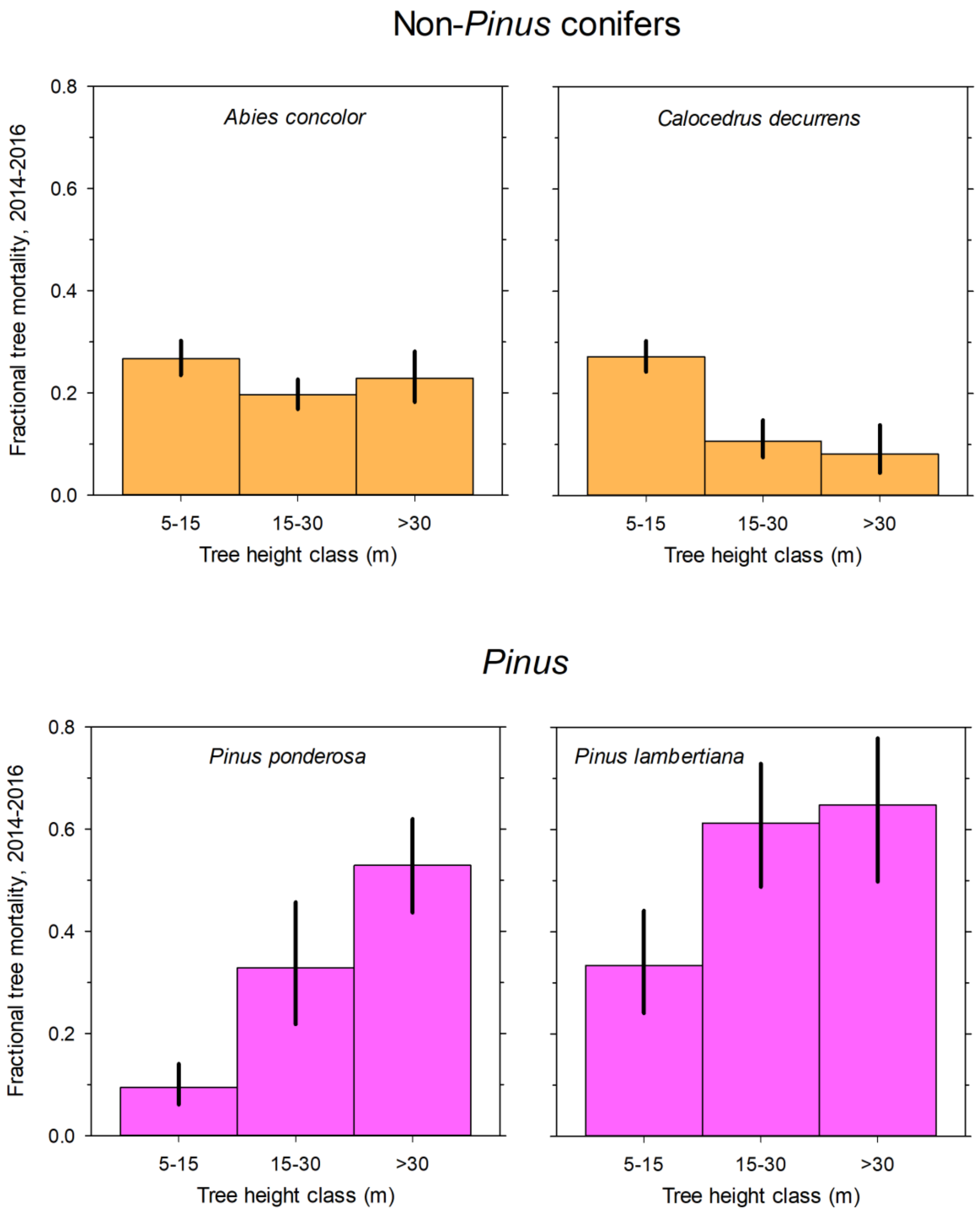

Supplementary Fig. 1 (continued from the preceding page)

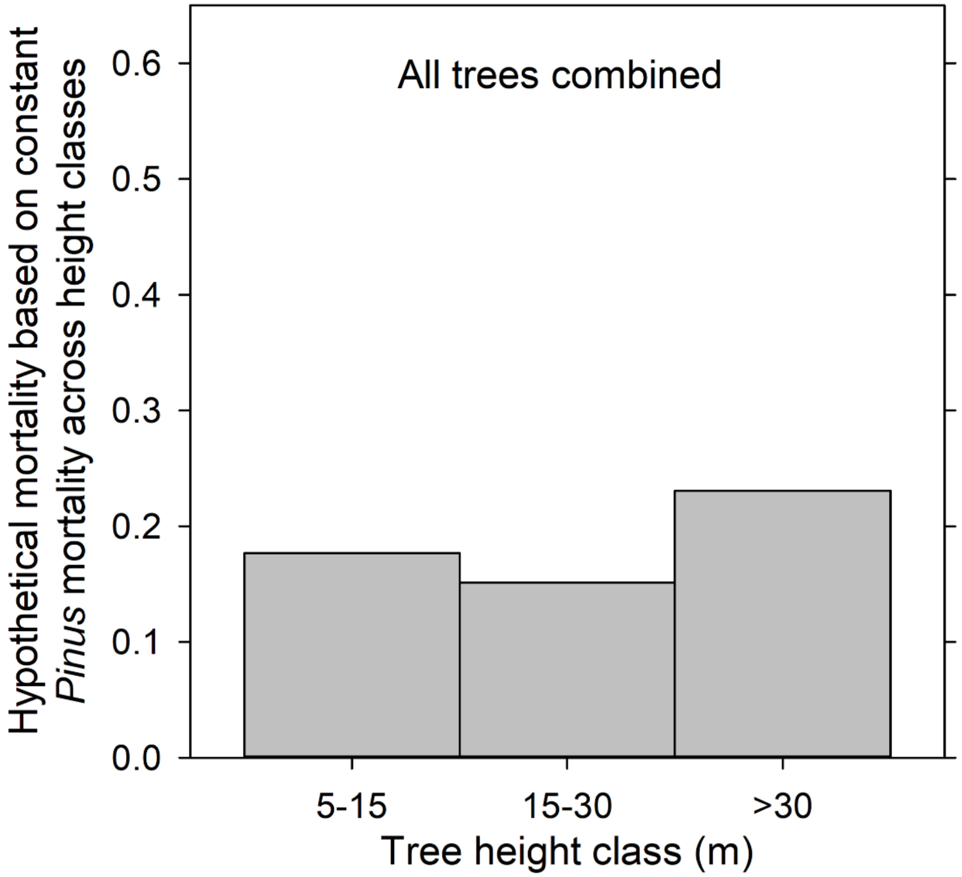

Supplementary Fig. 2: Hypothetical 2014-2016 mortality for all trees combined, calculated using actual relative abundances of taxonomic groups within height classes (Fig. 1c) and actual height- and taxa-specific mortality values for angiosperms and non-*Pinus* conifers (Fig. 1b), but assuming constant *Pinus* mortality (that of the *Pinus* population as a whole) across the three *Pinus* height classes (Supplementary Table 5). Because these results are hypothetical, no credible intervals are shown.

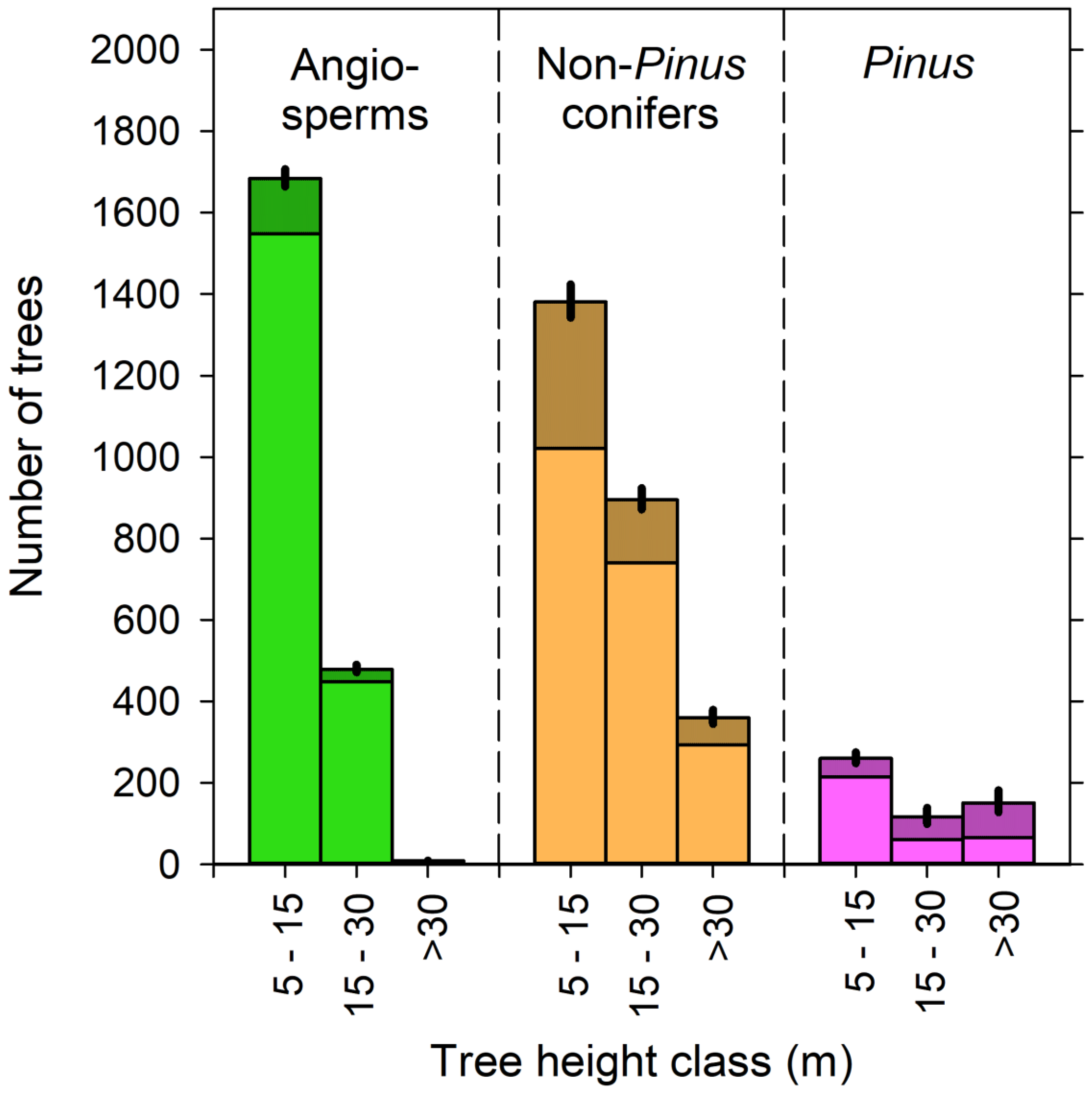

Supplementary Fig. 3: Numbers of trees in our 1705-ha study landscape, by taxonomic group, height class, and drought mortality status. Raw numbers are given in Supplementary Table 6. Gray shading at the tops of the bars represents numbers of trees that were estimated to be alive in 2013 but that died in 2014-2016 (with 95% credible intervals), calculated using Equations 6 and 7. As described in Methods, values of $m_{i,t}$ used in Equation 6 were derived from the posterior distributions of parameters estimated from 45,000 Markov Chain Monte Carlo iterations (three 15,000-iteration chains). Unshaded parts of the bars show actual numbers of living trees recorded in 2016. Thus, total bar heights represent numbers of trees estimated to have been alive in 2013 (numbers differ from those in Supplementary Table 3, which represent all living and dead trees in our sample regardless of year of death). Small trees dominate, as is typical for Sierra Nevada forests[11], but in contrast to Stovall et al.'s data[13] (see Supplementary Note 4).

Supplementary References

1. Safford, H. D. & Stevens, J. T. *Natural Range of Variation for Yellow Pine and Mixed Conifer Forests in the Sierra Nevada, Southern Cascades, and Modoc and Inyo National Forests, California, USA*. General Technical Report PSW-GTR-256 (Albany, California, U.S. Department of Agriculture, Forest Service, Pacific Southwest Research Station, 2017). 229 pages. https://www.fs.usda.gov/treesearch/pubs/55393
2. Cocking, M. I., Varner, J. M. & Engber, E. A. in *Proceedings of the Seventh California Oak Symposium: Managing Oak Woodlands in a Dynamic World* (tech. coords. Standiford, R. B. & Purcell, K. L.) 505-514 (General Technical Report PSW-GTR-251, Berkeley, California, U.S. Department of Agriculture, Forest Service, Pacific Southwest Research Station, 2015). https://www.fs.usda.gov/treesearch/pubs/50018
3. van Mantgem, P. J., Stephenson, N. L., Keifer, M. & Keeley, J. Effects of an introduced pathogen and fire exclusion on the demography of sugar pine. *Ecol. Appl.* **14,** 1590-1602 (2004).
4. Maloney, P. E., Vogler, D. R., Eckert, A. J., Jensen, C. E. & Neale, D. B. Population biology of sugar pine (*Pinus lambertiana* Dougl.) with reference to historical disturbances in the Lake Tahoe Basin: implications for restoration. *For. Ecol. Manage.* **262,** 770-779 (2011).
5. Stephenson, N. L. *Climatic control of vegetation distribution: The role of the water balance with examples from North America and Sequoia National Park, California*. Dissertation, Cornell University, Ithaca, New York (1988).
6. Fites-Kaufman, J. A., Rundel, P., Stephenson, N. & Weixelman, D. A. in *Terrestrial Vegetation of California* (eds M. Barbour, M., Keeler-Wolf, T. & Schoenherr, A. A.) 456-501 (University of California Press, 2007).
7. Stephenson, N. L., Das, A. J., Ampersee, N. J., Bulaon, B. M. & Yee, J. L. Which trees die during drought? The key role of insect host-tree selection. *J. Ecol.* **107,** 2383-2401 (2019).
8. Fettig, C. J., Mortenson, L. A., Bulaon, B. M. & Foulk, P. B. Tree mortality following drought in the central and southern Sierra Nevada, California, U.S. *For. Ecol. Manage.* **432,** 164-178 (2019).
9. Pile, L. S., Meyer, M. D., Rojas, R., & Roe, O. in *Proceedings of the 19th Biennial Southern Silvicultural Research Conference* (compiler Kirschman, J. E.) 89-96 (e-General Technical Report SRS- 234, Asheville, North Carolina, U.S. Department of Agriculture, Forest Service, Southern Research Station, 2018). https://www.fs.usda.gov/treesearch/pubs/57284
10. Restaino, C., *et al.* Forest structure and climate mediate drought-induced tree mortality in forests of the Sierra Nevada, USA. *Ecol. Appl.* **29**, e01902 (2019).
11. Young, D. J. N., *et al*. Forest recovery following extreme drought in California, USA: natural patterns and effects of pre-drought management. *Ecol. Appl.*, e02002 (2019). (https://doi.org/10.1002/eap.2002)

12. Pile, L. S., Meyer, M. D., Rojas, R., Roe, O. & Smith, M. T. Drought impacts and compounding mortality on forest trees in the southern Sierra Nevada. *Forests* **10**, 237 (2019). (doi:10.3390/f10030237)
13. Stovall, A. E., Shugart, H. & Yang, X. Tree height explains mortality risk during an intense drought. *Nature Comm.* **10,** 4385 (2019). (https://doi.org/10.1038/s41467-019-12380-6)
14. Das, A., Battles, J., van Mantgem, P. J. & Stephenson, N. L. Spatial elements of mortality risk in old-growth forests. *Ecology* **89**, 1744-1756 (2008).
15. Das, A., Battles, J., Stephenson, N. L. & van Mantgem, P. J. The contribution of competition to tree mortality in old-growth coniferous forests. *Forest Ecology and Management* **261**, 1203-1213 (2011).
16. Gersonde, R., Battles, J. J. & O'Hara, K. L. Characterizing the light environment in Sierra Nevada mixed-conifer forests using a spatially explicit light model. *Can. J. For. Res.* 34, 1332-1342 (2004).
17. Westman, W. E. Aboveground biomass, surface area, and production relations of red fir (*Abies magnifica*) and white fir (*A. concolor*). *Can. J. For. Res.* 17, 311-319 (1987).
18. Barrett, T. M. Optimizing efficiency of height modeling for extensive forest inventories. *Can. J. For. Res.* 36, 2259-2269 (2006).
19. Garman, S. L., Acker, S. A., Ohmann, J. L. & Spies, T. A. Asymptotic height-diameter equations for twenty-four tree species in western Oregon. *Research Contribution 10*, Forest Research Laboratory, Oregon State University, Corvallis, Oregon (1995). 22 pages. https://ir.library.oregonstate.edu/dspace/bitstream/1957/7655/1/rc10.pdf
20. Keyser, C. E. (compiler). *Inland California and Southern Cascades (CA) Variant Overview – Forest Vegetation Simulator*. Internal Report, U.S. Department of Agriculture, Forest Service, Forest Management Service Center, Fort Collins, Colorado (2008, revised 25 September 2018). 79 pages. https://www.fs.fed.us/fmsc/ftp/fvs/docs/overviews/FVSca_Overview.pdf
