# Supplementary material for: Height-related changes in forest composition explain increasing tree mortality with height during an extreme drought
Source: Nat Commun. 2020 Jul 7;11:3402. doi: 10.1038/s41467-020-17213-5 (PMC7341764; doi:10.1038/s41467-020-17213-5)
Supplement: Supplementary file 3 — Reporting Summary [file 41467_2020_17213_MOESM3_ESM.pdf]

nature research

Corresponding author(s): Nathan L. Stephenson

Last updated by author(s): 2020/05/18

Reporting Summary

Nature Research wishes to improve the reproducibility of the work that we publish. This form provides structure for consistency and transparency in reporting. For further information on Nature Research policies, see Authors & Referees and the Editorial Policy Checklist.

Statistics

For all statistical analyses, confirm that the following items are present in the figure legend, table legend, main text, or Methods section.

n/a	Confirmed	
☐	☒	The exact sample size (*n*) for each experimental group/condition, given as a discrete number and unit of measurement
☐	☒	A statement on whether measurements were taken from distinct samples or whether the same sample was measured repeatedly
☒	☐	The statistical test(s) used AND whether they are one- or two-sided<br>*Only common tests should be described solely by name; describe more complex techniques in the Methods section.*
☐	☒	A description of all covariates tested
☒	☐	A description of any assumptions or corrections, such as tests of normality and adjustment for multiple comparisons
☐	☒	A full description of the statistical parameters including central tendency (e.g. means) or other basic estimates (e.g. regression coefficient) AND variation (e.g. standard deviation) or associated estimates of uncertainty (e.g. confidence intervals)
☒	☐	For null hypothesis testing, the test statistic (e.g. *F*, *t*, *r*) with confidence intervals, effect sizes, degrees of freedom and *P* value noted<br>*Give P values as exact values whenever suitable.*
☐	☒	For Bayesian analysis, information on the choice of priors and Markov chain Monte Carlo settings
☒	☐	For hierarchical and complex designs, identification of the appropriate level for tests and full reporting of outcomes
☒	☐	Estimates of effect sizes (e.g. Cohen's *d*, Pearson's *r*), indicating how they were calculated

Our web collection on statistics for biologists contains articles on many of the points above.

Software and code

Policy information about availability of computer code

Data collection	Field data were collected in Microsoft Access (version Microsoft Access 2016) using Microsoft Access forms.
Data analysis	Analyses were performed using R 3.6.2 with the coda 0.19.3, rjags 4.10, and R2jags 0.5.7 packages in combination with JAGS 4.3.0 software.

For manuscripts utilizing custom algorithms or software that are central to the research but not yet described in published literature, software must be made available to editors/reviewers. We strongly encourage code deposition in a community repository (e.g. GitHub). See the Nature Research guidelines for submitting code & software for further information.

Data

Policy information about availability of data

All manuscripts must include a data availability statement. This statement should provide the following information, where applicable:

- Accession codes, unique identifiers, or web links for publicly available datasets
- A list of figures that have associated raw data
- A description of any restrictions on data availability

The data are available in the ScienceBase repository, https://doi.org/10.5066/P99RNGXH.

Field-specific reporting

Please select the one below that is the best fit for your research. If you are not sure, read the appropriate sections before making your selection.

☐ Life sciences ☐ Behavioural & social sciences ☒ Ecological, evolutionary & environmental sciences

Ecological, evolutionary & environmental sciences study design

All studies must disclose on these points even when the disclosure is negative.

Study description	Within a low-elevation forest of California's southern Sierra Nevada, we sought to characterize and interpret size- and species-specific tree mortality rates during California's extreme 2012-2016 drought. Our forested study landscape was selected because it (1) spanned the forested elevational band that suffered greatest tree mortality in the southern Sierra Nevada, (2) was relatively easily accessible, and (3) occurred in the general vicinity of several additional long-term forest monitoring plots that had detailed data on agents of tree mortality.
Research sample	Our sample was meant to represent the population of living and dead trees within a low-elevation (1524-1829 m [5000-6000 feet]), 1705-ha forested landscape in the southern Sierra Nevada, California, USA, with the ultimate goal of characterizing and interpreting size- and species-specific tree mortality rates during California's extreme 2012-2016 drought. Our forested study landscape was selected because it (1) spanned the forested elevational band that suffered greatest tree mortality in the southern Sierra Nevada, (2) was relatively easily accessible, and (3) occurred in the general vicinity of several additional long-term forest monitoring plots that had detailed data on agents of tree mortality. The subset of trees analyzed in the present study (trees >5 m tall) comprised 5855 living and dead trees belonging to 15 species.
Sampling strategy	Sample locations were selected a priori using Generalized Random Tessellation sampling (GRTS), which provides a spatially balanced sample that has a true probability design, allowing valid inference for the entire 1705-ha study area (Stevens & Olsen 2004, Journal of the American Statistical Association 99(465):262-278). Although sample locations were selected a priori, exact sample size was not. Rather, our past experience statistically analyzing tree mortality rates in the southern Sierra Nevada led us to set a broad goal of sampling several thousand trees within at least 50 0.1 ha plots. We thus used GRTS to define a core set of 50 plot locations to visit. The GRTS approach also allows creation of a set of "oversampling" plots -- which are to be sampled in consecutive order -- beyond the initial 50; we defined 100 potential oversampling plots. As data were collected, starting at about 20 plots we periodically checked to see if newly-added plots were fundamentally changing the size- and species-specific mortality rates we were calculating (e.g., as presented in Fig. 3 of Stephenson et al. 2019, Journal of Ecology 107:2383-2401). Little change occurred with added plots beyond the first 30 to 40 plots. However, we took advantage of available funds and field personnel by collecting additional (oversampling) plots beyond our initial goal of 50, until the field season ended in late October, at which time we had 89 plots.
Data collection	Plot locations were selected a priori, as described under "Sampling strategy." In the field, plot centers were determined using a proximity alarm on handheld GPS units, with the alarm sounding when proximity to the predetermined GRTS location was within the measurement error of the device. Plots were then defined as the area within a 17.84 m horizontal radius (determined by electronic rangefinders) from these plot centers. High-precision plot center locations were then determined using a JAVAD Triumph-2 GPS unit that, after post-processing, determined actual locations with sub-meter accuracy. Established plot centers usually were close to the predetermined GRTS targets; for example, our original GRTS targets fell within established plot boundaries 93% of the time. Within each plot, two or more experienced field personnel used electronic tablets to record each standing tree by (1) species, (2) trunk diameter at breast height (DBH; breast height = 1.37 m) by 5-cm classes, and (3) condition (living or dead). All standing conifers >0 cm DBH and all standing angiosperms ≥5 cm DBH were recorded, where "standing" was defined as any tree leaning less than 45 degrees from vertical and at least 1.37 m in length. Each dead tree was further classified according to its foliage and fine twig retention, to allow us to estimate year of death (as described in Stephenson et al. 2019, Journal of Ecology 107:2383-2401). Data were collected by N. Ampersee, B. Borden, A. Das, K. DeChain, C. Loria, D. Murphy, J. Nelson, B. Permar, A. Pfaff, N. Stephenson, and A. Young, with occasional assistance from other experienced personnel.
Timing and spatial scale	Data were collected from 15 June 2016 through 26 October 2016, with most samples later in the period. Specifically, 4, 14, 22, 27, and 22 plots were sampled in June, July, August, September, and October of 2016, respectively. Samples were within our predefined 1705-ha landscape.
Data exclusions	Crews attempted to establish 98 plots, but 6 of the predetermined plot locations could not, in the judgment of field personnel, be reached safely (this was a predefined exclusion criterion -- e.g., for dangerously steep cliffs), and 3 plots overlapped roads (another predefined exclusion criterion), leaving 89 plots that were sampled. None of the 89 sampled plots was excluded from analysis.
Reproducibility	Plot locations were meant to be reproducible from high-precision plot center locations, determined using a JAVAD Triumph-2 GPS unit that, after post-processing, determined actual locations with sub-meter accuracy.
Randomization	As described in more detail under "Sampling strategy," plot locations were selected a priori using Generalized Random Tessellation sampling (GRTS), which provides a spatially balanced sample that has a true probability design, allowing valid inference for the entire 1705-ha study area (Stevens & Olsen 2004, Journal of the American Statistical Association 99(465):262-278).
Blinding	Blinding was not necessary (or possible), because no treatments were applied and field personnel collected purely observational data with no a priori hypotheses in mind.

Did the study involve field work? ☒ Yes ☐ No

Field work, collection and transport

Field conditions	Field work was conducted in the summer and autumn of 2016, as weather allowed (e.g., personnel did not work during rainstorms or when wind might cause branches or trees to fall).
Location	The 1705-ha study landscape spanned 1524-1829 m elevation (5000-6000 feet) within parts of the Marble Fork of the Kaweah

Location	River and Yucca Creek watersheds of Sequoia National Park, California, USA, UTM coordinates 336000-343000 East, 4045000-4052000 North (Zone 11, NAD 83).
Access and import/export	No samples were collected or transported. All work was approved by the U.S. National Park Service through a research permit issued (revised) on 2 May 2016.
Disturbance	Travel to plots was by foot. This observational study caused no meaningful disturbances.

Reporting for specific materials, systems and methods

We require information from authors about some types of materials, experimental systems and methods used in many studies. Here, indicate whether each material, system or method listed is relevant to your study. If you are not sure if a list item applies to your research, read the appropriate section before selecting a response.

Materials & experimental systems

n/a	Involved in the study	
☒	☐	Antibodies
☒	☐	Eukaryotic cell lines
☒	☐	Palaeontology
☒	☐	Animals and other organisms
☒	☐	Human research participants
☒	☐	Clinical data

Methods

n/a	Involved in the study	
☒	☐	ChIP-seq
☒	☐	Flow cytometry
☒	☐	MRI-based neuroimaging
